# Experimental Investigations on Microstructure, Properties and Wear Behavior of Chopped Basalt Fiber and Molybdenum Disulfide Reinforced Epoxy Matrix Composites

**DOI:** 10.3390/polym17101371

**Published:** 2025-05-16

**Authors:** Santhosh Kumar P. C., Manickam Ravichandran, Vinayagam Mohanavel, Nachimuthu Radhika

**Affiliations:** 1 Department of Mechanical Engineering, K. Ramakrishnan College of Engineering, Tiruchirappalli 621112, Tamil Nadu, India; santhoshpckrce@gmail.com; 2 Centre for Sustainable Materials Research, Department of Mechanical Engineering, Academy of Maritime Education and Training (AMET) Deemed to be University, Kanathur, Chennai 603112, Tamil Nadu, India; mohanavel2k18@gmail.com; 3 Department of Mechanical Engineering, Amrita School of Engineering, Amrita Vishwa Vidyapeetham, Coimbatore 641112, Tamil Nadu, India; n_radhika1@cb.amrita.edu

**Keywords:** polymer composites, basalt fiber, molybdenum disulfide, compression molding, properties

## Abstract

This study examined the impact of molybdenum disulfide (MoS_2_) addition as a filler in epoxy composites reinforced with chopped basalt fibers (CBF), maintaining the basalt fiber content at a constant 40 wt. %. The investigation focused on physical, microstructural, mechanical, and sliding-wear properties. Testing revealed that tensile, impact, compressive, and flexural strengths improved with MoS_2_ content from 0 to 8 wt. %. However, at 12 wt. % loading, these properties declined due to uneven dispersion and particle agglomeration. An increase in hardness was observed with rising MoS_2_ content, with a maximum value of 98 HV at 16 wt. %. Wear testing was conducted using a Taguchi L16 orthogonal array, evaluating the effects of multiple parameters. The results indicated that MoS_2_ content had the most significant influence on wear rate (WR), followed by applied load (P) and sliding distance (D), while sliding velocity (V) had minimal impact on specific wear rate (SWR) and coefficient of friction (COF). Scanning electron microscopy (SEM) was used to analyze wear mechanisms, and analysis of variance (ANOVA) confirmed the optimal conditions.

## 1. Introduction

The emphasis of industries today is on the satisfaction of consumer needs and being able to produce goods which are acceptable as regards to the environment. In this decade, polymer materials have been increasingly used in place of metal materials because they exhibit lower friction coefficients and can bear loads as well [1,2]. The majority of the most advanced structural matrix composites combine epoxy with micro-fibers such as carbon, glass, Kevlar, and/or boron filaments, as well as fillers such as SiC, Al_2_O_3_, B_4_C, and carbon nanotubes, in particular in aviation. Research, including the study by Neves et al. [3], highlights the extensive use and high demand for epoxies across various sectors such as engineering, structural applications, commercial industries, and military aerospace.

In an effort to reduce their negative effects on the environment, natural fibers are increasingly being used as reinforcement in polymer composites. A notable example is basalt rock, a black or gray material formed through the rapid cooling of basaltic lava [4]. Basalt fibers, derived from this rock, were first developed between 1953 and 1954 by the Moscow Research Institute of Glass and Plastic, with large-scale production initiated in 1985 at a fiber laboratory in Ukraine [5]. Understanding how the chemical composition of basalt affects its melting point during manufacturing helps improve both production efficiency and product uniformity. This is because physical properties such as melting temperature, crystallization temperature, and strength are all determined by the material’s chemical composition. Basalt fibers have many benefits such as high strength and modulus, high elongation at break, high thermal and chemical stability (−200 to 600–800 °C), thermal insulation, and weather resistance, easy processing, and are eco-friendly and cost effective, thus making them ideal substitutes for other reinforcements like glass and carbon fibers [6,7,8,9,10]. These characteristics are particularly beneficial in the context of creating polymer composites that are sustainable, environmentally friendly, and lightweight.

Composites reinforced with basalt fibers have remarkable mechanical qualities that are on par with synthetic fibers and provide an environmentally responsible substitute. Their incorporation enhances polymer composites with improved mechanical, thermal, and tribological performance [11,12].

Several studies have examined enhancements in basalt fiber composites through nanofillers. Graphene nanoparticles showed optimal wear resistance at 0.3 wt. % [13], while nano/micro silica [14,15] and TiC nanoparticles [16] improved hardness, impact strength, and wear resistance. Basalt particle addition improved flexural and tensile strength by ~8% and ~15%, respectively [17]. However, research on wear behavior with basalt fillers remains limited [18,19,20], despite fly ash additions showing mechanical benefits [21].

Desai et al. [22] investigated glass fiber composites and observed that nanoclay/fly ash-filled glass epoxy composites exhibited superior tensile and impact strength, whereas zinc oxide/fly ash-filled glass epoxy composites demonstrated excellent flexural strength, particularly at a 60 wt. % filler concentration. The composites can be produced by various methods, including compression molding, suction infusion, hand layup, injection molding, extrusion, injection molding, and filament winding (Omran et al., 2021) [23]. Among all the technologies, compression molding stands out, with its lower cost and advantages such as improved product quality, stability, repeatability, smooth surface, mechanical properties, and resistance to flame (Li et al., 2020; Jose et al., 2022; Dani et al., 2012; Park et al., 2012; Kim et al., 2019) [24,25,26,27,28]. The parameters chosen for this study include different weights of MWCNT/SiO_2_ filler (0%, 1%, 2.9%, and 3%) and gradually decreasing the weight percentage of the epoxy matrix (40%, 39%, 38%, and 37%) with compression pressures ranging from 5 MPa to 35 MPa, and molding temperatures between 40 °C and 100 °C. These materials were fabricated using hand lay-up and compression molding techniques. The tensile strength was found to increase to 184.38 MPa when the molding temperature was optimized, and the wear weight lowered to 95.06 mg/kg when the filler content was reduced [29].

Similarly, optimal impact resistance was observed in jute-reinforced polypropylene composites under specific molding parameters [30].

Tribological studies have shown varied effects of fillers: PTFE and nano Al_2_O_3_ modified wear and friction in basalt fabrics [31,32], alumina reduced wear while basalt increased friction [33], and sliding speed was a key factor in red mud–polyester composites [34]. Hybrid textile composites with WC fillers also benefited from optimized mixing using the Taguchi method [35].

Polymer composites filled with nano- and micro-particles have been widely studied for their enhanced mechanical and tribological performance. However, most studies have focused on synthetic fibers or unidirectional fiber composites, with limited attention paid to chopped basalt fiber (CBF)-reinforced systems combined with solid lubricant fillers like molybdenum disulfide (MoS_2_). MoS_2_, known for its excellent lubricating properties and thermal stability, has the potential to significantly reduce wear and improve frictional behavior when used as a filler in composites. Also, hybrid epoxy composites and the impact of compression molding settings on their mechanical and tribological properties have received less attention. In order to close this gap, the current study investigates the tribological and mechanical characteristics of epoxy composites supplemented with MoS_2_ as a filler and 40% chopped basalt fiber. Mechanical properties were evaluated using tensile, flexural, impact, hardness, and wear tests. Under dry sliding conditions, the tribological performance of epoxy resin reinforced with basalt fiber was also examined. The Taguchi technique was used to determine how wear parameters affected the specific wear rate (SWR) and coefficient of friction (COF).

## 2. Materials and Methods

**Figure 1:**
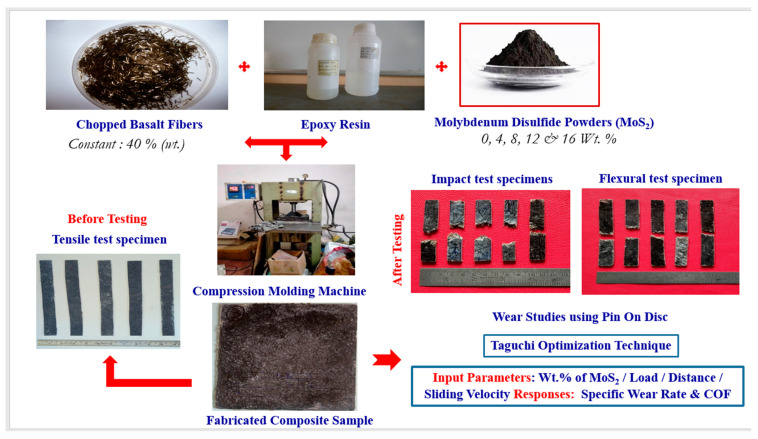
Experimental plan, sample preparation, and testing.

Epoxies are known for their excellent resistance to water and heat with high corrosion resistance [36,37]. In the proposed composites, epoxy resin was used as the matrix material, combined with an amine-based hardener (K-6 hardener) in a 1:1 ratio. Reinforcement was provided by basalt fiber (40 wt. %) and molybdenum disulfide (MoS_2_), incorporated in varying amounts of 0, 4, 8, 12, and 16 wt. %. The basalt fiber, with an average density of 2.67 g/cm^3^, was procured from CF Composites Ltd., Delhi, India, and processed into 20 mm lengths and 10 μm diameters using a standard cutting knife. The composite samples were prepared through a compression molding process. A compression molding machine with upper and lower metallic dies was employed to produce samples with dimensions of 200 mm × 120 mm × 6 mm. The MoS_2_ filler was combined with the epoxy resin in predetermined weight percentages and rotated at a steady 55 rpm for 15 min to guarantee even dispersion. After achieving a homogeneous mixture, basalt fibers were added at a fixed content of 40 wt. %. This mixture was then placed in the molding machine and subjected to compression to fabricate the samples. In order to assess the impact of MoS_2_ filler and basalt fibers on the epoxy matrix’s characteristics, four composite formulations were created, each with a fixed 40 weight percent basalt fiber content and a different MoS_2_ filler percentage. The experimental plan for this study is outlined in Figure 1.

SEM examination was performed using a ZEISS SIGMA SEM instrument to examine the morphology of the manufactured composite samples. Additionally, Energy-Dispersive X-ray Analysis (EDAX) was performed to verify that the composites contained the necessary constituents. Theoretical calculations are made to determine the density of chopped basalt fiber and MoS_2_ reinforced epoxy composites (0, 4, 8, 12, and 16 weight percent). The Archimedes method was employed to find the experimental density in accordance with ASTM D792-13 [38]. The average experimental density of the basalt composites and the mixture’s void ratio were calculated by analyzing five samples containing the same weight ratio [38].

Vickers microhardness testers are used for microhardness testing. A diamond indenter with a square base and a 136° angle between opposing faces (pyramid shape) was used to take measurements. According to ASTM E384-17 [39], a load of 22.52 N was put on for 20 s. To obtain the average hardness value, each specimen had five indentations made at various locations. An impact test was used to find the ability of the specimen under study to withstand sudden impact load. In this study, the impact resistance of the formed basalt fiber composites were evaluated using an Izod impact test rig. This method determines the kinetic energy needed to initiate and propagate a fracture until the specimen material breaks, in accordance with ASTM D4812 [40], the standard for the Izod test. An upright test specimen, held vertically in place by grippers, received kinetic energy from an air pendulum throughout testing.

Tensile tests were performed using a UTE-40 universal testing machine at a speed of 2 mm/min. Both ends of the specimens were clamped into machine clamps for the uniaxial tensile test, and a controlled speed and progressively increasing stress were applied until failure. The average results of testing five specimens from each composite formulation were recorded. During the flexural test, tensile and compressive stresses were generated within the specimen, causing shear stresses along its centerline as the beam bent under the applied load. Following the ASTM D790 [41] standard for nominal specimens, this test was conducted using a universal testing machine with a 400 kN capacity. A load was applied to the specimens at a rate of 4 mm/min until they broke, with the specimens resting on two supports that were 75 mm apart. The load at the failure point was then used to compute the flexural stress.

Compressive strength (CS), which evaluates a material’s ability to withstand deformation under load, was measured according to ASTM D3410 standard [42]. Care was taken to ensure that fractures occurred at the desired locations, as the results’ significance depended on their placement. Specimens were clamped at both ends, and compression was applied as the machine’s jaws moved closer together. The force applied was recorded relative to changes in the gauge length. The compression test was conducted using a universal testing machine that had a maximum capacity of 400 kN and applied a loading rate of 4 mm/min.

To analyze the wear characteristics of MoS_2_- and chopped basalt fiber-reinforced epoxy composites with weight fractions of 0, 4, 8, and 12%, a pin-on-disk machine was utilized for two body abrasive tests using the ASTM G-99 standard [43]. The test samples used were 30 mm long and 10 mm square. Before the test, cotton tissue soaked in acetone was used to wipe the specimens and the disk surfaces, and it was allowed to air dry. An electronic balance was used to weigh the pin assembly with a 0.1 mg precision.

The tests were conducted at sliding velocities of 1.0, 1.5, 2.0, 2.5 m/s and 5.0, 10, 15, and 20 N loads. The weight loss was recorded at 500, 750, 1000, and 1250 abraded distances. An L16 orthogonal array was selected for this investigation, with Table 1 detailing the input parameters and their levels.

## 3. Results and Discussion

### 3.1. SEM Analysis

**Figure 2:**
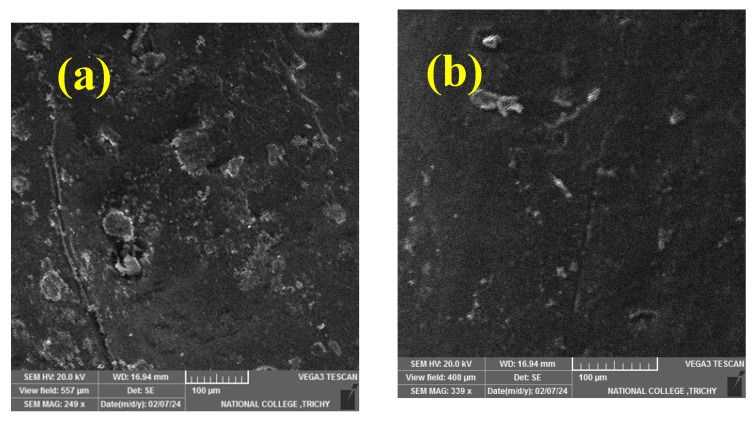

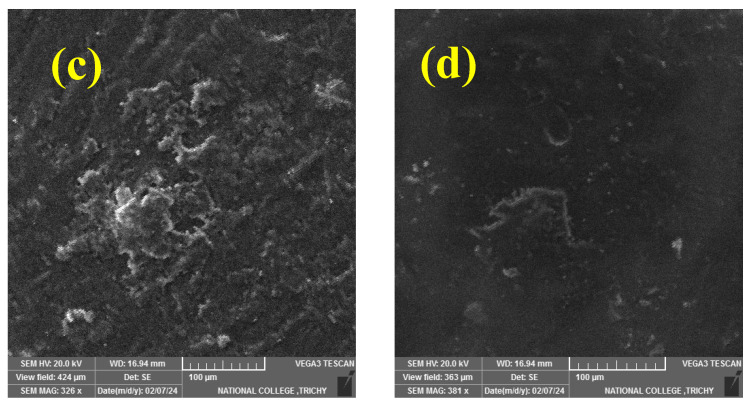
(**a**–**d**). SEM images (**a**) 40% CBF + 4% MoS_2_; (**b**) 40% CBF + 8% MoS_2_; (**c**) 40% CBF + 12% MoS_2_; (**d**) 40% CBF + 16% MoS_2_.

Figure 2a–d presents SEM micrographs of chopped basalt fibers and MoS_2_-reinforced epoxy composites with varying concentrations of MoS_2_ particles. Figure 2a–c illustrate a uniform distribution of basalt and MoS_2_ within the epoxy. However, in Figure 2d, agglomeration due to the increased MoS_2_ content is evident. These agglomerations lead to porosity in the composites, particularly noted at the composition of 40% CBF and 16% MoS_2_, where higher viscosity in the matrix contributes to this issue. Beyond the threshold limit of reinforcements, the likelihood of agglomeration and void formation increases due to poor dispersion, as discussed in references [11,44,45].

### 3.2. EDAX Analysis

**Figure 3:**
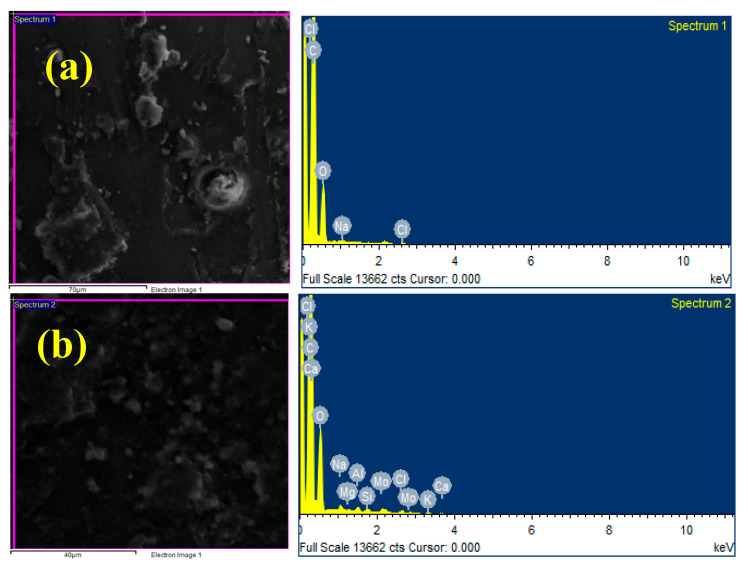

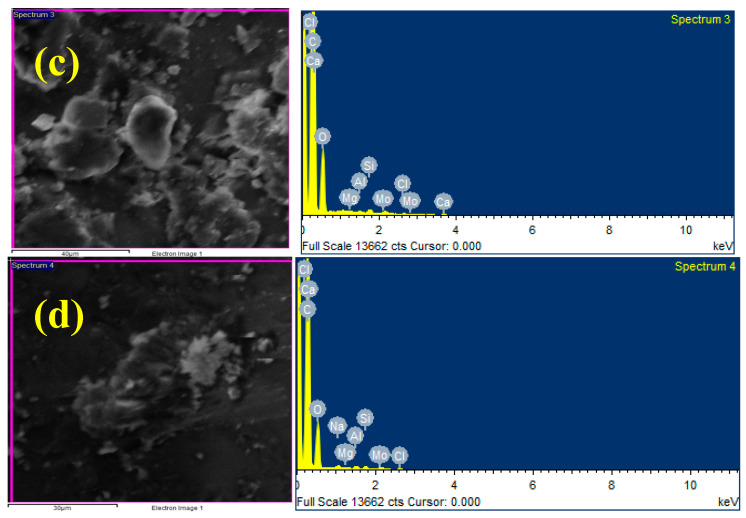
EDAX of (**a**) 40% CBF + 4% MoS_2_; (**b**) 40% CBF + 8% MoS_2_; (**c**) 40% CBF + 12% MoS_2_; (**d**) 40% CBF + 16% MoS_2_.

Figure 3a–d displays the EDAX images for the composites: Figure 3a: 40% CBF + 4% MoS_2_; Figure 3b: 40% CBF + 8% MoS_2_; Figure 3c: 40% CBF + 12% MoS_2_; and Figure 3d: 40% CBF + 16% MoS_2_. The EDAX images show that the composites contain epoxy resin and reinforcing elements such as silicon, potassium, magnesium, calcium, aluminum, and molybdenum. The intensity of MoS_2_ in the matrix is high for the composite sample contain 16% of MoS_2_ which is undoubtedly evident in Figure 3d.

### 3.3. Physical Properties

**Figure 4:**
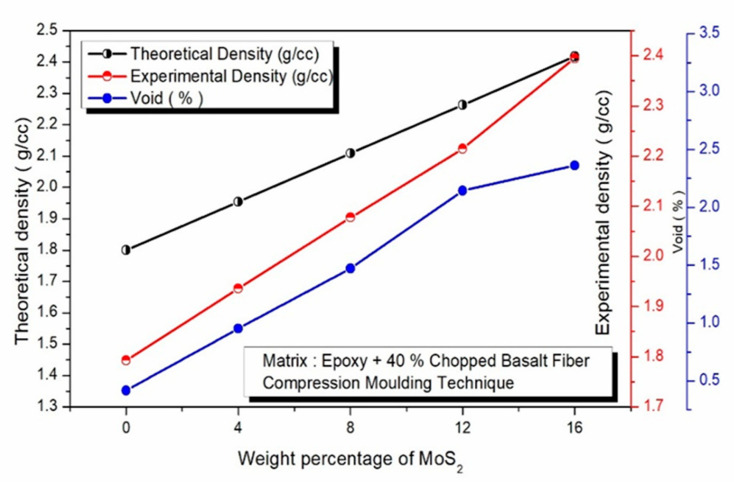
Effect of MoS_2_ in epoxy–40% CBF composite on density and void (%).

The effect of MoS_2_ on the void content and density of epoxy composites supplemented with 40% chopped basalt fiber (CBF) is shown in Figure 4. The composite density consistently increases with the addition of MoS_2_ and chopped basalt fiber. However, due to the presence of voids within the nanocomposites, the experimentally measured density falls short of the theoretical values. Additionally, Figure 4 highlights the void percentage in epoxy composites containing chopped basalt fiber and MoS_2_, emphasizing the critical influence of voids on the overall performance of these materials. Although voids in nanocomposites are typically unavoidable, employing advanced fabrication techniques can help minimize their formation. Among the composites, the epoxy matrix on its own exhibits the lowest void fraction. However, introducing MoS_2_ into the epoxy matrix with 40% chopped basalt fiber results in a higher void fraction compared to composites reinforced solely with chopped basalt fiber. This increase in void content correlates directly with the MoS_2_ concentration, as higher filler levels tend to encourage void generation. The incorporation of MoS_2_ into the composite matrix was observed to influence the void content significantly. As the MoS_2_ content increased, a corresponding rise in the percentage of voids was detected. This behavior can be attributed to two important factors. One primary cause is the tendency of MoS_2_ particles to agglomerate at higher concentrations, leading to non-uniform dispersion within the matrix. These agglomerates can disrupt the flow of the matrix material during processing, creating microstructural inconsistencies that trap air and result in void formation. Furthermore, the presence of MoS_2_ alters the interfacial compatibility between the filler and the matrix, and such interfacial issues can facilitate void nucleation during processing. As noted by Agarwal et al. [46], composites are categorized by void fraction (Vf) into three quality levels: excellent (Vf < 1%), good (Vf between 1% and 5%), and defective (Vf > 5%). The void fraction of the composites evaluated in this study ranges from 1% to 2.5%, indicating that they fall within the “good” quality classification.

### 3.4. Mechanical Properties

**Figure 5:**
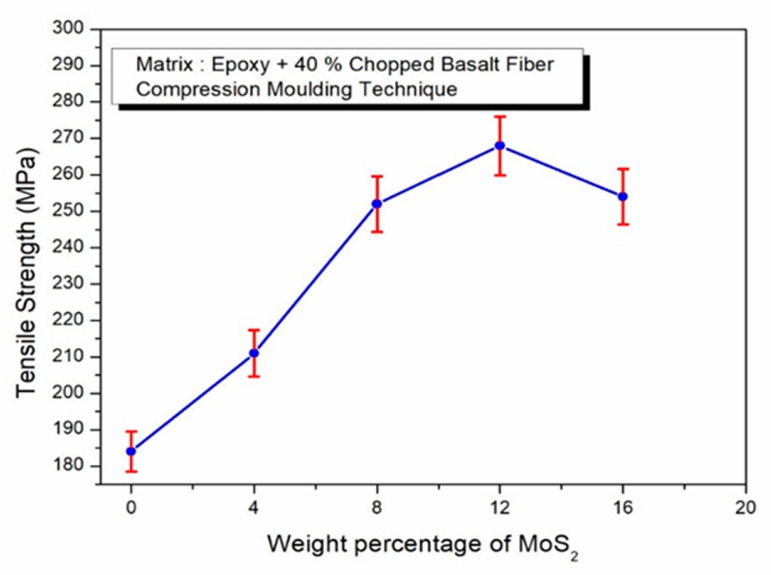
Effect of MoS_2_ in epoxy–40% CBF composite on tensile strength.

Figure 5 illustrates the tensile strength of epoxy composites reinforced with 40% chopped basalt fiber (CBF) and varying concentrations of MoS_2_. The results reveal the effect of MoS_2_ particles on the composite’s tensile properties. The highest tensile strength values recorded were 184 MPa, 211 MPa, 252 MPa, and 268 MPa for composites containing 40% basalt fiber and 0%, 4%, 8%, and 12% MoS_2_ by weight, respectively. The homogeneous dispersion of MoS_2_ particles throughout the epoxy matrix and basalt fiber is responsible for the observed increase in tensile strength with up to 12% MoS_2_ reinforcement. The tensile strength enhancement can be attributed to the synergistic interaction between MoS_2_ particles and the epoxy matrix, where MoS_2_ acts as a nano-scale stress distributor and filler, enhancing the interfacial adhesion between the epoxy and the discontinuous basalt fibers. Similar hybrid reinforcement effects have been reported in short-fiber composites incorporating graphene or ceramic nanoparticles studies, where excessive filler loading led to agglomeration and interfacial stress concentrations. Moreover, since chopped fibers depend on effective stress transfer through the matrix, MoS_2_ enhances load transfer across discontinuous fibers. However, the tensile strength decreases to 254 MPa when the MoS_2_ content is raised to 16 weight percent. According to Ozsoy et al. [47], the ideal reinforcing levels for AlO_3_ and TiO_2_ fillers in epoxy composites were approximately 2.5 weight percent; larger concentrations resulted in decreased tensile strength. This drop is consistent with their findings. Excess filler content, typically in the range of 4–5%, can lead to localized dispersion and particle agglomeration, weakening the interfacial bonding and leading to the reduced tensile strength of the composite. During tensile testing, specimens fractured at the gauge length, exhibiting a fiber pull-out failure mode. Initially, the fibers bore the applied load; however, once the fibers’ load-bearing capacity was exceeded, the load transferred to the matrix. The fiber and matrix’s interfacial connection was weakened by this change, which ultimately led to the composite’s fracture.

**Figure 6:**
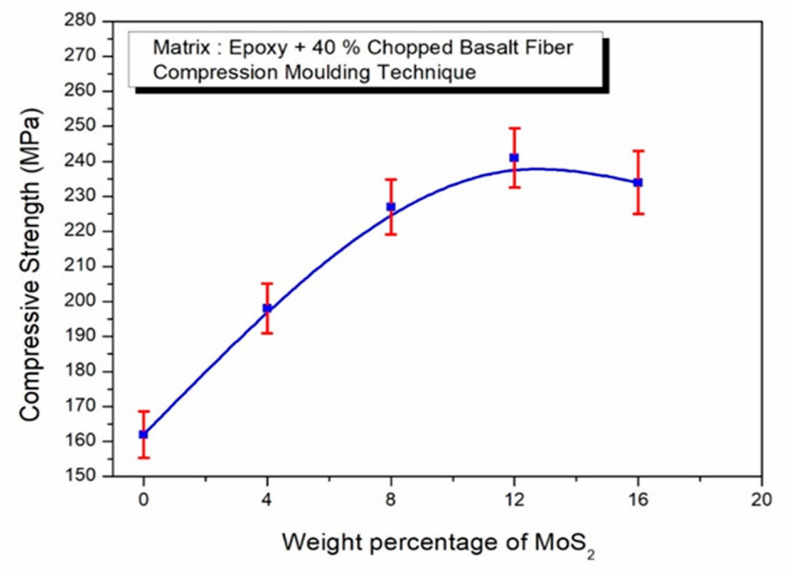
Effect of MoS_2_ in epoxy–40% CBF composite on ‘CS’.

The ‘CS’ of the epoxy–40% CBF-MoS_2_ composite is shown in Figure 6. The composite’s ‘CS’ was raised by the addition of fibers and MoS_2_, both of which have a high hardness. The maximum ‘CS’ achieved by the fabricated composites was 241 MPa. During the compressive tests, all components underwent plastic deformation, with the hard basalt fibers embedding into the matrix. Prolonged compression allows these fibers to resist loads without undergoing plastic deformation. The transfer of load was achieved by the strong adhesive interaction between basalt fibers and epoxy which enhances the resistance of the composite to compression. The increase in the weight percentage of MoS_2_ improves the ‘CS’ up to 12% and then it decreases. The reason is that the increase in MoS_2_ particle lead to agglomeration and poor mixing in the matrix.

**Figure 7:**
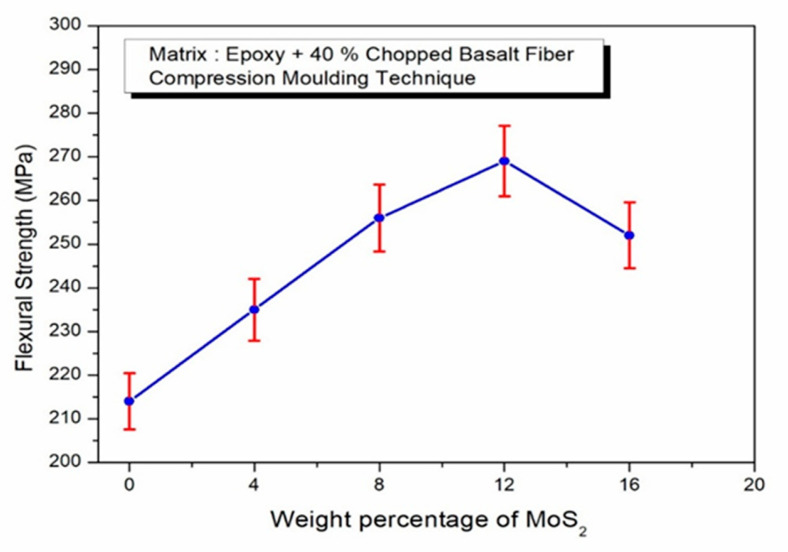
Effect of MoS_2_ in epoxy–40% CBF composite on flexural strength.

The result suggest that the increase in MoS_2_ within the composites significantly improves the flexural strength values, although there is a limit which, once exceeded, has a negative effect on the strength-absorbing qualities, particularly when MoS_2_ exceeds 12 wt. %. As shown in Figure 7, the epoxy sample without fillers achieved the minimum flexural strength, while the sample containing 12 wt. % MoS_2_ demonstrated the highest improvement in strength. The inclusion of chopped basalt fibers and MoS_2_ fillers is responsible for these variances in flexural strength. Flexural strength is increased as a result of the even distribution of MoS_2_ particles and the chopped basalt fibers in the epoxy resin matrix. The improved interfacial adhesion between the fibers and resin facilitates the more effective transfer of mechanical stress, which is the main cause of this improvement. The fillers play a crucial role in occupying void spaces, which further strengthens the bond between the matrix and reinforcement, ultimately leading to better performance under flexural stress. In contrast, the lower flexural strength of the sample without MoS_2_ reinforcements is likely due to the lack of effective particle reinforcement.

**Figure 8:**
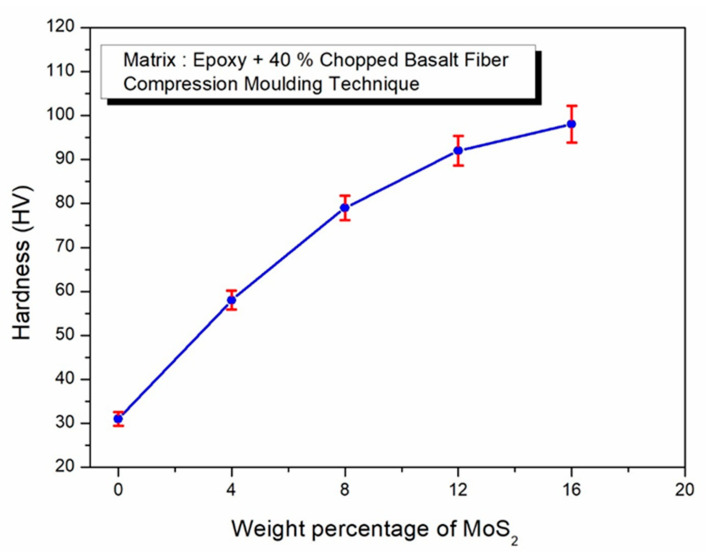
Effect of MoS_2_ in epoxy–40% CBF composite on hardness.

The influence of MoS_2_ on the hardness property of the epoxy composites reinforced with 40% chopped basalt fiber (CBF) is depicted in Figure 8. Hardness measures a material’s ability to resist indentation or scratching. The hardness of unreinforced epoxy is 25 HV, but it increases to 31 HV when 40 wt. % basalt fibers are incorporated into the epoxy. This increase in hardness can be ascribed to the even dispersion of chopped basalt fibers in the epoxy matrix, which fortifies the fiber–epoxy link and improves the composite’s resistance to the indenter’s compressive force. When MoS_2_ is added, the hardness also increases again to achieve the highest at 98 HV when 16 wt. % MoS_2_ is used. The change in MoS_2_ particles to high density in the polymer matrix caused the polymer-based structure to be tight and compact. Similar findings by Raajeshkrishna et al. [48] highlight the role of outer basalt layers in glass/basalt sandwich hybrid composites, demonstrating how they significantly influence the hardness of such materials. The increased hardness observed here enhances the composites’ ability to resist scratching and wear, making them suitable for high-wear applications.

**Figure 9:**
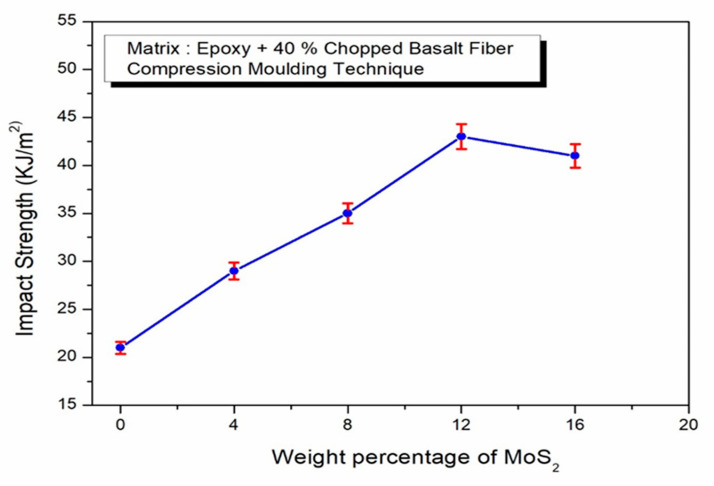
Effect of MoS_2_ in epoxy–40% CBF composite on impact strength.

Impact strength behavior in basalt fiber-reinforced epoxy composites with MoS_2_ particles at varying weight percentages (0, 4, 8, 12, and 16 wt. %) is shown in Figure 9. It is noted that the impact strength of these composites rises up to 12 wt. % before decreasing at 16 wt. %, which aligns with findings from tensile, compressive, and flexural assessments. This effect can be attributed to the reduced void content in basalt fiber composites, which stems from improved interfacial bonding between the fibers and the matrix. This enhanced bonding strengthens the composite’s ability to endure impact loads effectively. The recorded impact strength indicates that the particulates are well-distributed throughout the epoxy matrix, effectively dissipating impact energy. Consequently, chopped basalt fiber-reinforced epoxy composites show notable enhancements in overall mechanical strength characteristics.

### 3.5. Wear Studies Using Taguchi Design

In this study, the MoS_2_ weight percentage (0, 4, 8, and 12 wt. %) was considered the key material design parameter, while ‘P’ (applied load), ‘V’ (sliding velocity), and ‘D’ (sliding distance) were included as representative operational conditions. To systematically assess the influence of these factors and to identify the optimal configuration for minimizing wear, the Taguchi orthogonal design (L16 array) was employed. In this study, each test specimen was assigned a coded designation based on the levels of the four parameters (A: MoS_2_ wt. %, B: load (P); C: sliding speed (V); and D: sliding distance (D). Given that mechanical properties declined beyond 12 wt. % MoS_2_ addition, composites with 16 wt. % MoS_2_ were excluded from the wear study. The objective function was converted to an S/N ratio in the Taguchi method to analyze ‘WR’. The S/N ratios and mean values were computed using Minitab 17 software, with results presented in Table 2. The S/N ratio response table and main effects plots were then used to determine the optimal parameter values for these control factors.

The lowest SWR and COF were observed under the following parameters: 12 wt. % MoS_2_, a ‘P’ of 20 N, a ‘V’ of 1.0 m/s, and a ‘D’ of 1000 m (A4B4C1D3), resulting in a ‘SWR’ of 0.8169 mm^3^/m. This demonstrates that the composite with a higher MoS_2_ content exhibits the lowest ‘WR’. The increased MoS_2_ content enhances particle movement resistance within the epoxy matrix, leading to a reduction in ‘WR’. S/N ratios were calculated for both COF and SWR to analyze these effects.

**Figure 10:**
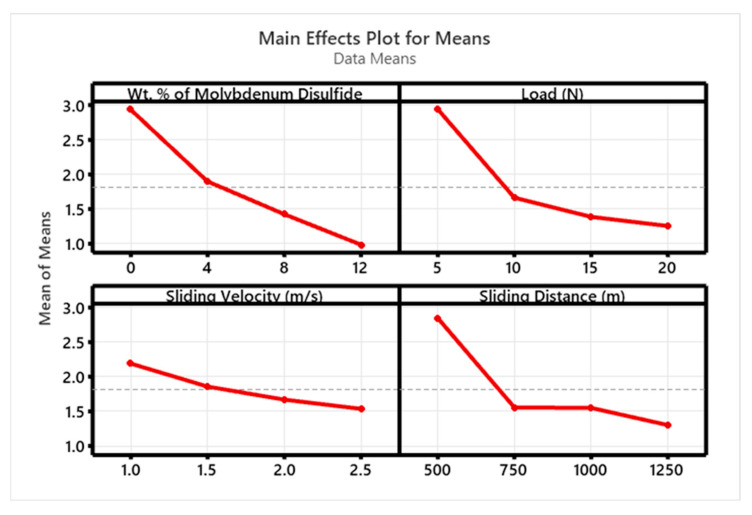
SN ratio plot for SWR.

**Figure 11:**
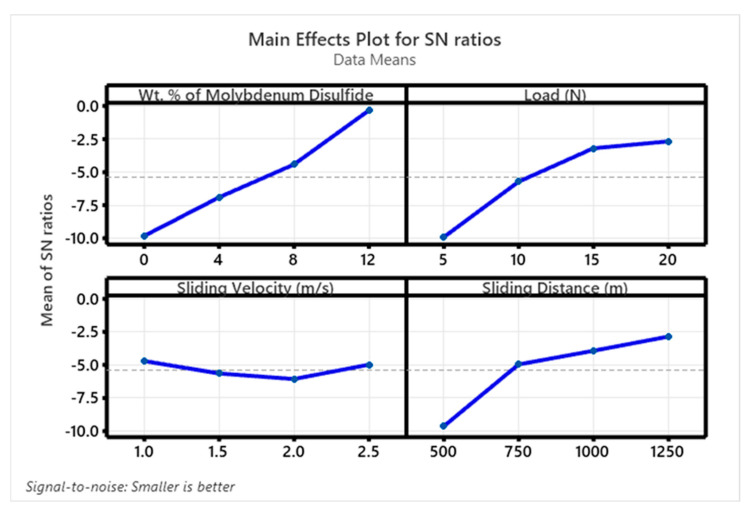
Mean plot for SWR.

**Figure 12:**
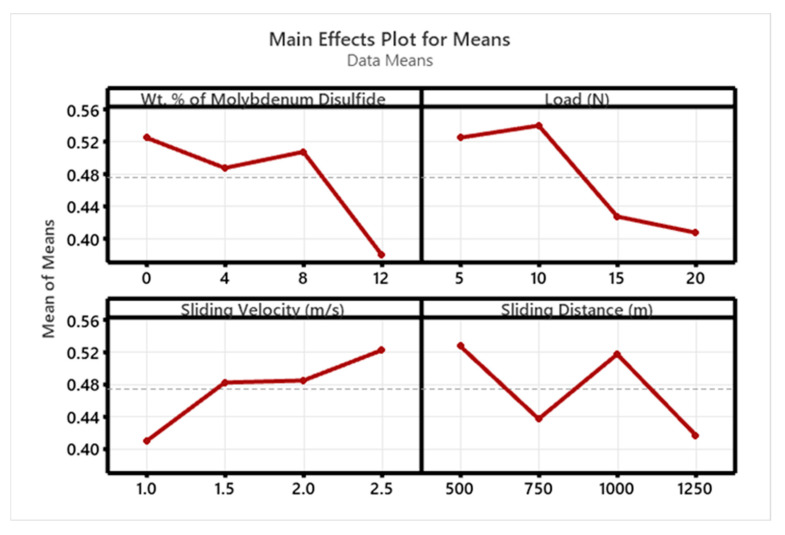
SN ratio plot for COF.

**Figure 13:**
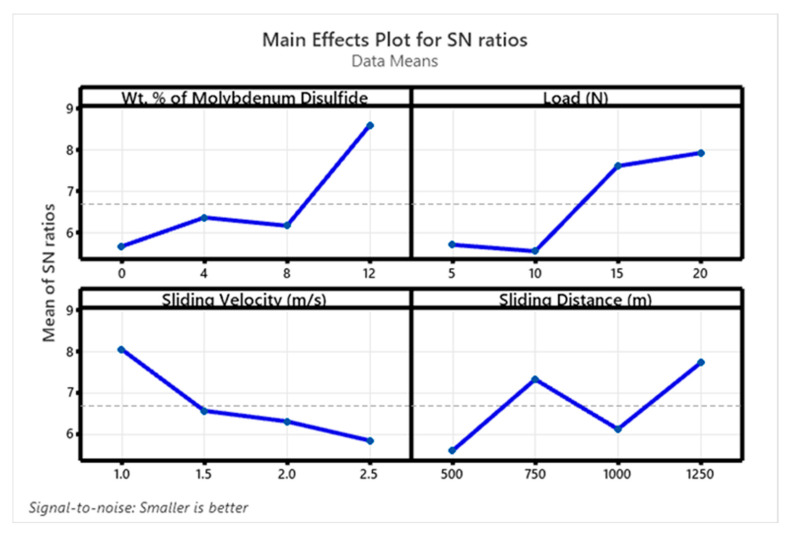
Mean plot for COF.

Figure 10 and Figure 11 display the S/N ratio and mean plots for SWR, while Figure 12 and Figure 13 show these plots for the coefficient of friction (COF). Based on the S/N ratio plots, the optimal conditions for SWR are A4B4C1D4 (12 wt. % MoS_2_, 20 N ‘P’, 1 m/s ‘V’, and 1250 m ‘D’, with A1B2C4D1 for COF (0 wt. % MoS_2_, 10 N ‘P’, 2.5 m/s ‘V’, and 500 m ‘D’). Increasing the MoS_2_ content decreases the SWR of the composite. The main effect plots in Figure 10 and Figure 11 indicate that higher MoS_2_ content notably decreases SWR. Additionally, both SWR and COF main effect plots suggest that ‘V’ has minimal impact on results. The “Smaller is Better” approach was used to assess process parameter effects on SWR and COF, with rankings for wt. % reinforcement, ‘P’, ‘V’, and ‘D’ detailed in Table 3, Table 4, Table 5 and Table 6.

**Figure 14:**
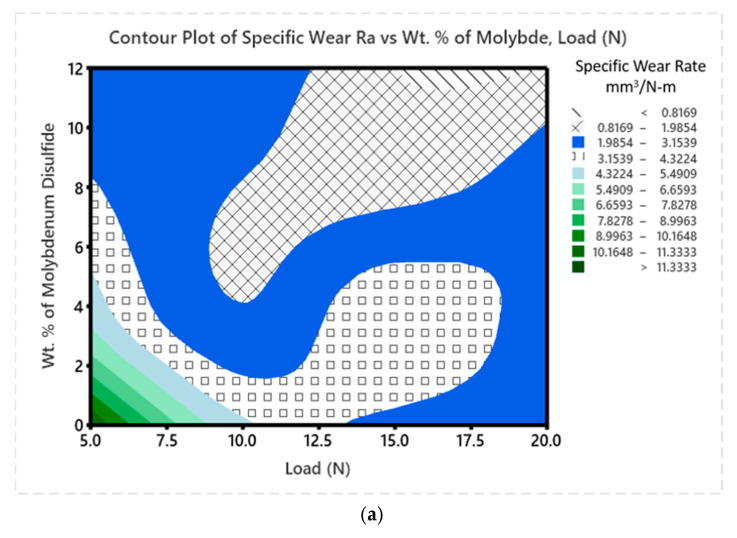

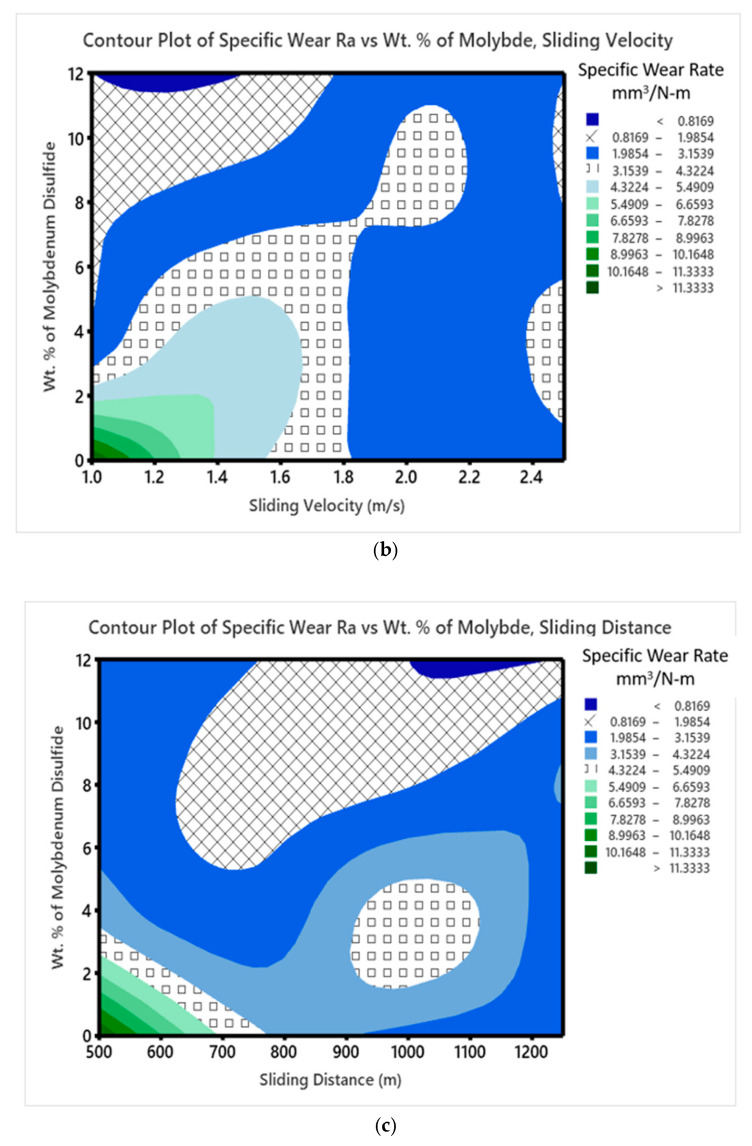

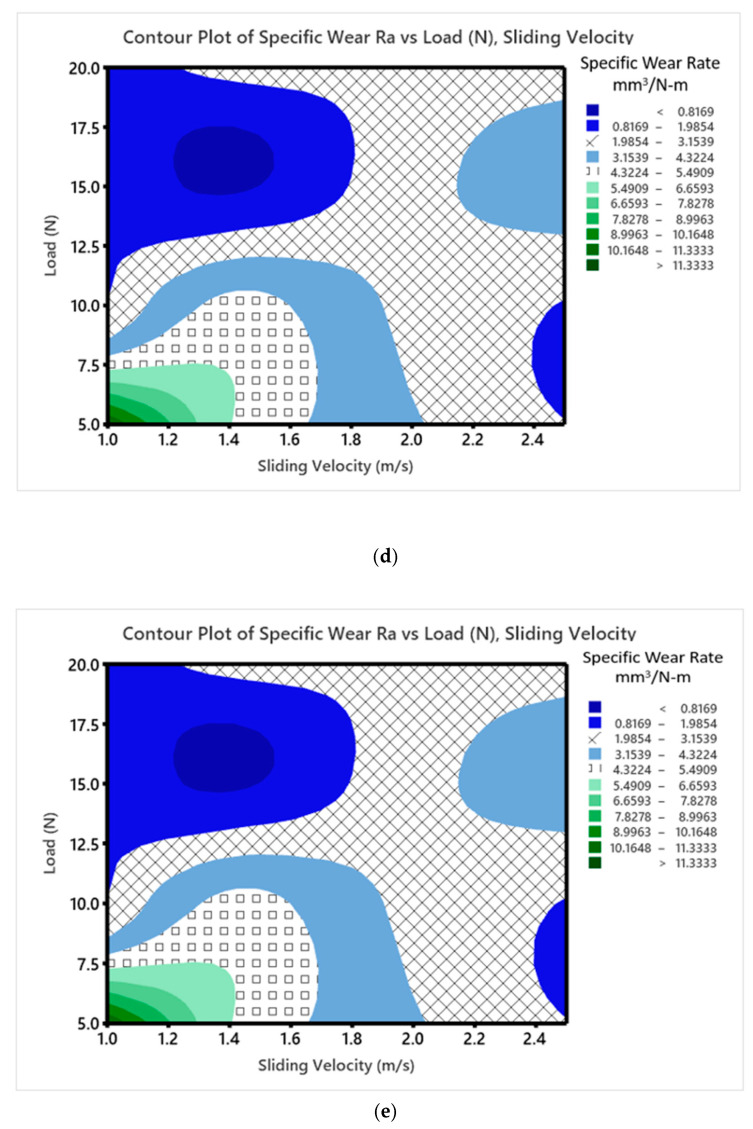

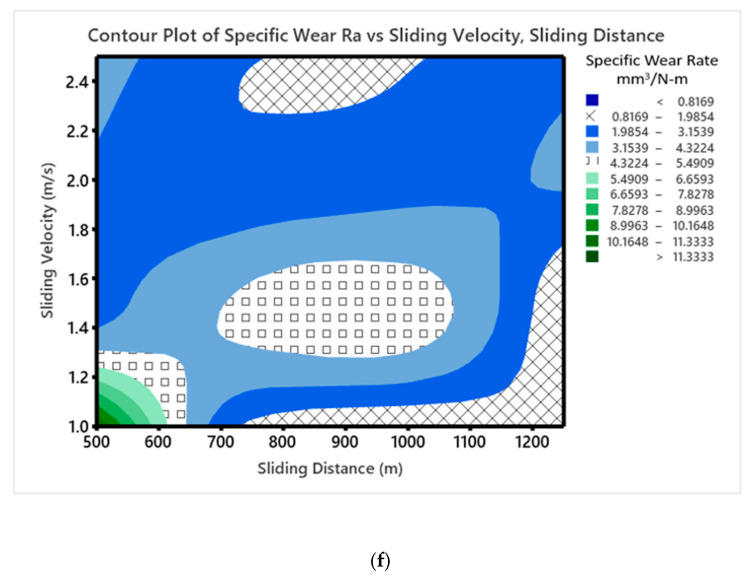
(**a**–**f**) Contour plot for SWR.

Figure 14a–f presents the contour plots for SWR. The plots in Figure 14a–c illustrate SWR variations with respect to ‘P’ and MoS_2_ wt. %, MoS_2_ wt. % and ‘V’, MoS_2_ wt. % and ‘D’, respectively. Across all these plots, the highest ‘WR’ occurs in the 0 wt. % MoS_2_ region, regardless of the other parameters. The lowest ‘WR’ are found in regions with 12 wt. % MoS_2_, suggesting that these composites possess enhanced wear resistance due to higher MoS_2_ content. Increased ‘P’ causes the wear of the composite matrix to rise, mostly as a result of the deformation of the material. Figure 14c,e,f show the contour plots for ‘D’ paired with MoS_2_ wt. %, ‘P’, and sliding distance, respectively. These plots confirm that a longer ‘D’ results in higher wear, likely due to heat generation at the contact surface between the pin and composite.

**Figure 15:**
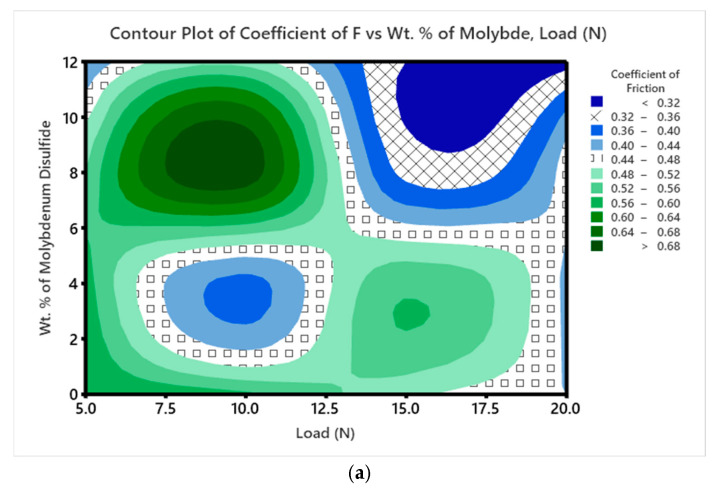

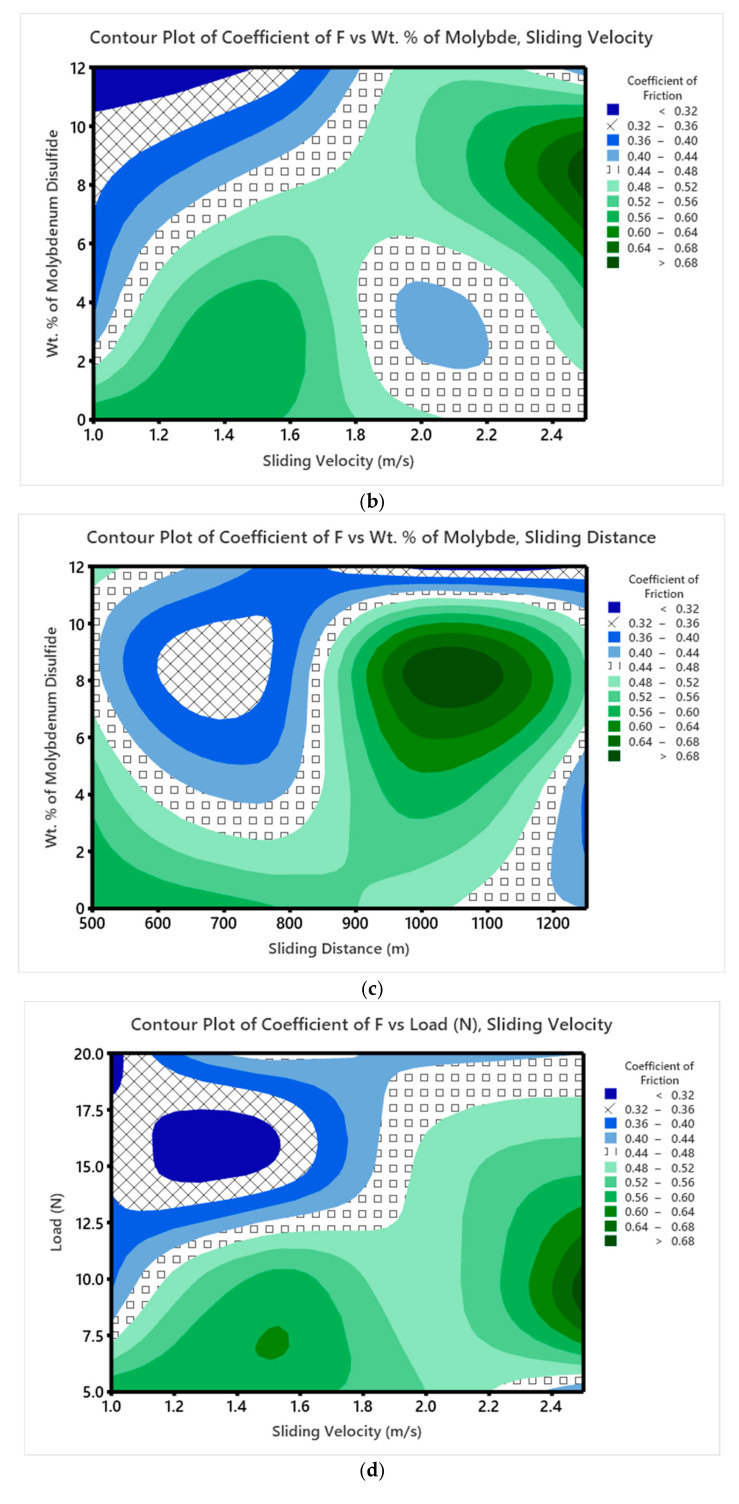

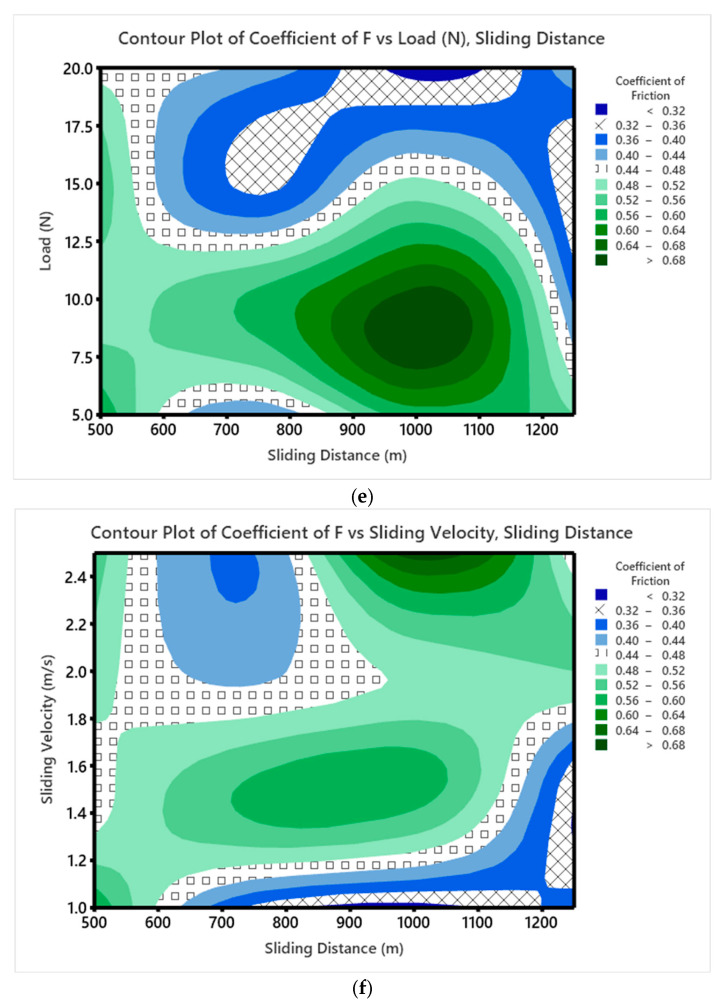
(**a**–**f**) Contour plot for COF.

Figure 15a–f presents the contour plots for the coefficient of friction (COF). Similarly to the trends observed for SWR, the lowest COF values occur in samples with higher MoS_2_ content. The contour plot in Figure 15f shows the relationship between COF, ‘V’, and ‘D’. As ‘V’ increases, the COF of the composite rises at longer ‘D’. Meanwhile, Figure 15e reveals that COF increases with ‘D’, even when the ‘P’ remains relatively unchanged.

**Figure 16:**
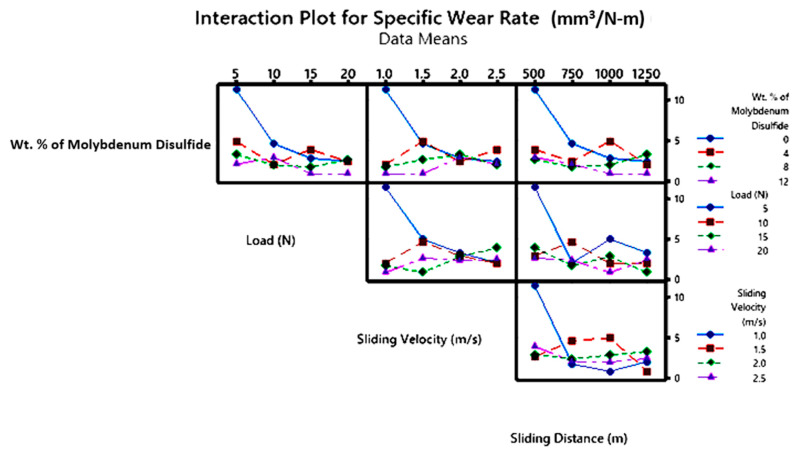
Interaction plot for SWR.

**Figure 17:**
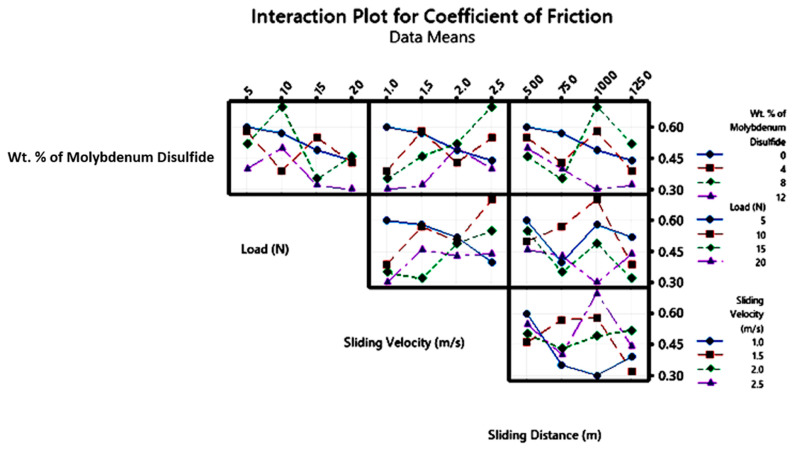
Interaction plot for COF.

An interaction plot illustrates the effects of interactions among variables, with lines representing each variable and its response. If lines are parallel, it indicates no interaction between the variables; if lines are non-parallel or intersect, statistical analysis confirms an interaction. Interaction plots for SWR and COF in Figure 16 and Figure 17 show that control factors significantly affect SWR and COF. The plot reveals that higher ‘WR’ is associated with lower MoS_2_ content and lower ‘P’. When considering MoS_2_ content in conjunction with other factors, it is evident that, at lower interaction levels, the epoxy matrix without MoS_2_ reinforcement exhibits the highest ‘WR’. The composite without MoS_2_ shows a notably higher ‘WR’, with a significant interaction effect from other factors. Wear is directly proportional to the applied normal pressure or ‘P’, as per Archard’s law, which suggests that ‘WR’ typically rises with ‘P’. The plot supports this relationship, showing that ‘P’ interacts significantly with other factors across various conditions.

ANOVA (analysis of variance) is a univariate statistical analysis used to decompose variability into systematic and random parts; in this case, the system factors were viewed as possessing a higher statistical weight than the random ones. Researchers have used ANOVA to test if and how the independent variables are impacting on the dependent variable using regression. This study assessed the effect of MoS_2_ percentage and wear parameters on SWR and COF through ANOVA test. The effects of factors influencing SWR and COF are given in Table 7 and Table 8, respectively. Their patterns explained residual variances which assisted in comparing different controlling parameters’ *p*-values attributed to error contributions. The smallest *p*-value corresponds to the greatest influence. The main driving forces for ‘SWR’ include the Wt. % of MoS_2_, ‘P’, and ‘D’, while ‘V’ does not impact SWR and COF significantly.

**Figure 18:**
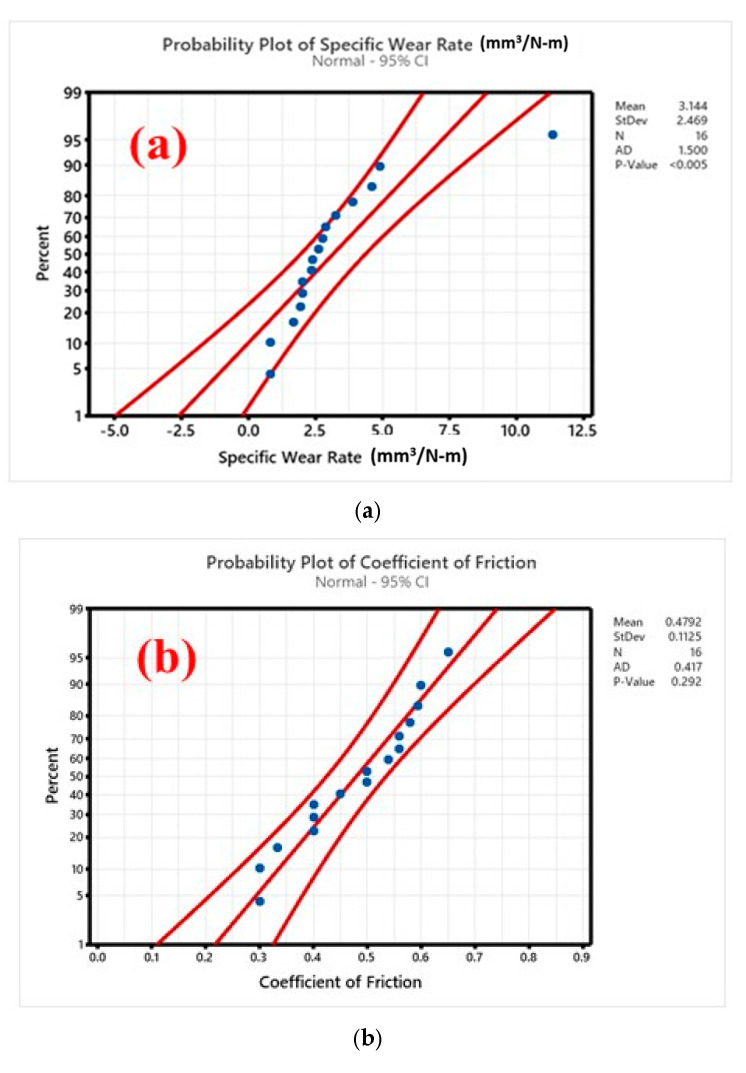
Probability plot for (**a**) SWR and (**b**) COF.

The equations relating SWR and COF were estimated from regression analysis. The relationships in the selected factors affecting ‘SWR’ are given in Equation (1), while Equation (2) deals with COF. Both ‘SWR’ and COF have normal residuals as normal probability plots, shown in Figure 18a and Figure 18b, respectively. The constructed points almost constitute a linear distribution around the limits of the confidence interval.

‘SWR’ (mm^3^/N.m) = 3.144 + 2.126 Wt. % of MoS_2__0 + 0.146 Wt. % of MoS_2__4
− 0.769 Wt. % of MoS_2__8 − 1.504 Wt. % of MoS_2__12 + 2.240 P (N)_5
− 0.286 P (N)_10 - 0.848 P (N)_15 - 1.105 P (N)_20 + 0.816 V (m/s)_1.0
+ 0.092 V (m/s)_1.5 − 0.324 V (m/s)_2.0 − 0.585 V (m/s)_2.5
+ 2.035 D (m)_500 − 0.479 D (m)_750 − 0.531 D (m)_1000
− 1.025 D (m)_1250
(1)

Coefficient of friction = 0.4750 + 0.0500 Wt. % of MoS_2__0 + 0.0125 Wt. % of
MoS_2__4
+ 0.0325 Wt. % of MoS2_8 - 0.0950 Wt. % of MoS2_12 + 0.0500 ‘P’ (N)_5
+ 0.0650 ‘P’ (N)_10 - 0.0475 ‘P’ (N)_15 - 0.0675 ‘P’ (N)_20
− 0.0650 V (m/s)_1.0 + 0.0075 V (m/s)_1.5 + 0.0100 V (m/s)_2.0
+ 0.0475 V (m/s)_2.5 + 0.0525 D (m)_500 − 0.0375 D (m)_750
+ 0.0425 D (m)_1000 - 0.0575 D (m)_1250
(2)

### 3.6. Worn Morphology

**Figure 19:**
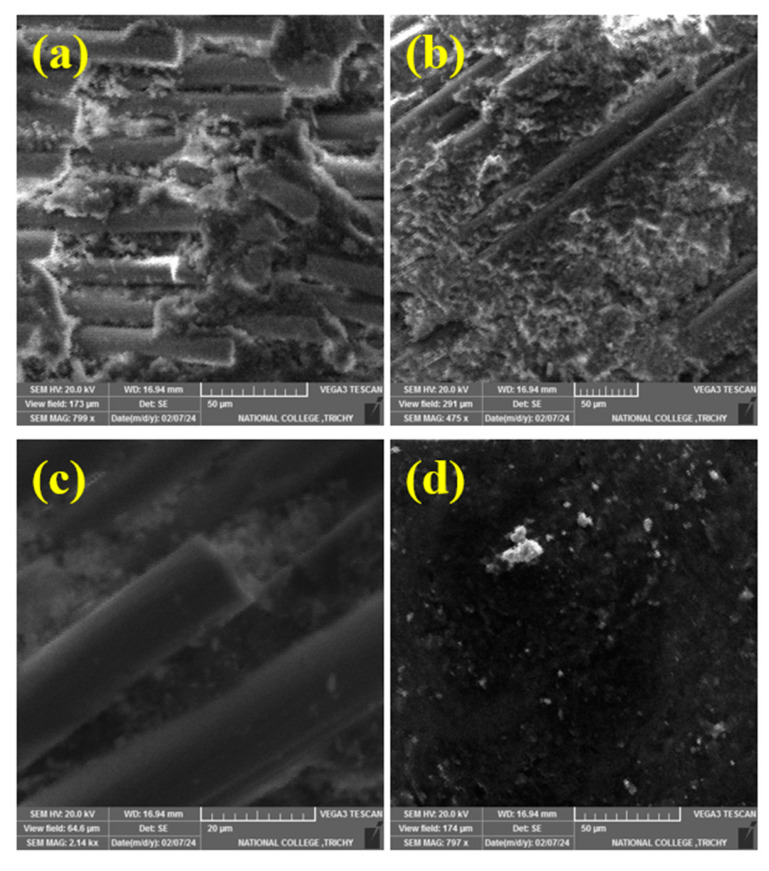
(**a**–**d**) Worn surfaces of epoxy–40% CBF–MoS_2_ composite.

**Figure 20:**
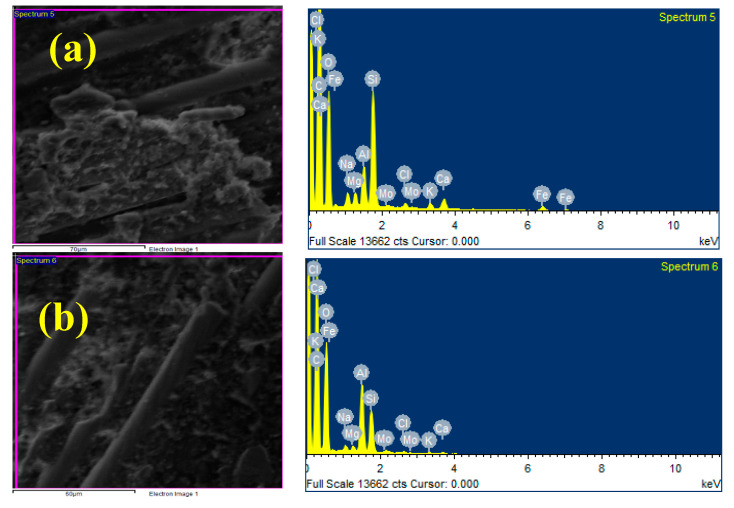

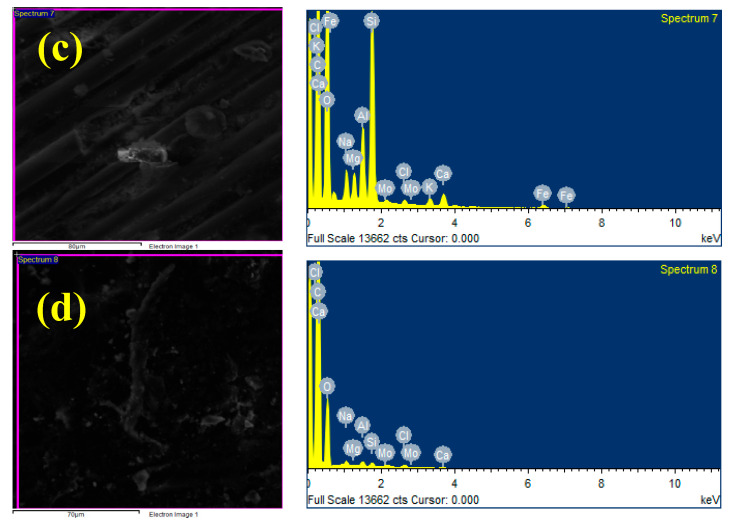
(**a**–**d**) Worn surfaces of epoxy–40% CBF–MoS_2_ composite.

Four phases make up the wear mechanism in polymer composites: matrix wear, reinforced fiber exposure, fiber breaking, and debris production. SEM observations of the wear-tested specimens confirm these mechanisms. Patches of resin and fiber breakage can be seen in Figure 19a,b. The epoxy matrix exhibits increased wear at the composite surface due to damage occurring between the fibers. Basalt fibers and epoxy provide a strong bonding ability that reduces fiber exposure and breakage in reinforced composites while also protecting the matrix. The phenomenon of fiber exposure and breakage is substantiated by Figure 19a,c. The high-roughness surface features of the composites scratch the surfaces and subsurfaces of the disks, leading to the volume loss resulting from cracking and the formation of pits in the matrix. In the end, these materials are lost in the form of debris, as in Figure 19d. When MoS_2_ particles were added, the composites with 12 weight percent of particles had better wear resistance. Figure 20a–d shows the EDAX of worn surfaces of Epoxy–40% CBF–MoS_2_ composites. The results of EDAX evidence the presence of epoxy resin, chopped basalt fibers, and MoS_2_ particles. Figure 20d shows the presence of elements like Mo, C, S, Cl, Ca, O, Na, Mg, Al, and Si, and these elements evidence the worn surface of the epoxy–40 wt. % CBF–12 wt. % MoS_2_ composite.

## 4. Conclusions

The current research focused on the effect of introducing MoS_2_ fillers on the mechanical properties and performance in the epoxy composites containing 40 wt. % chopped basalt fibers. The fabrication of composites was performed by compressive molding technique. The microstructures of the composites were studied using SEM and EDAX. After introducing MoS_2_ fillers into the composites, the specimens were further subjected to physical, mechanical, and wear tests. The main findings are presented below:

- Epoxy composites were obtained integrated with the chopped basalt fibers and MoS^2^ dispersed at the weight concentrations of 0, 4, 8, 12, and 16 wt. %.
- The addition of the MoS_2_ to the matrix was confirmed through SEM and EDAX, and the even dispersion of the protective MoS_2_ particles in the matrix was also confirmed.
- When MoS_2_ was added, the density of the composites rose; nevertheless, because of the voids in the composites, the experimental density was somewhat lower than the predicted density.
- The addition of 16 wt. % MoS_2_, when incorporated into the chopped basalt fibers, resulted in the highest hardness of 98 HV.
- Incorporating MoS_2_ fillers significantly enhanced the tensile strength of the composites, with the highest tensile strength recorded at 268 MPa for a 12% MoS_2_ addition, indicating effective stress and deformation resistance.
- The hardness of MoS_2_ and basalt fibers is responsible for the rise in compressive strength, which reached a peak of 241 MPa. During the compressive test, all components exhibited plastic deformation, with the hard basalt fibers embedding into the matrix.
- The investigation also found out that the MoS_2_ reinforcements delivered an enhancement of the flexural strength of the made composites. The MoS_2_-reinforced composites exhibited an improved flexural strength of about 269 MPa as compared to composites without fillers.
- It is also worth mentioning that the 16 wt. % MoS_2_ composite demonstrated reduced tensile, compressive, and flexural strengths as a consequence of a greater void fraction.
- To evaluate the wear characteristics of the composites, a L16 orthogonal array was deployed. The lowest ‘SWR’ and COF were registered at 0.8169 mm^3^/m at the loading conditions of 12 wt. %, 20 N ‘P’, 1.0 m/s velocity, and 1000 m sliding distance. High MoS_2_ concentrations minimized particle movement within the epoxy matrix, which lowered the ‘WR’, although ‘V’ only had a marginal impact.
- According to ANOVA results, the percentage of MoS_2_-reinforced weight had a greater impact on the composites’ ‘WR’ than the other components, with load and ‘D’ coming in second and third, respectively, while ‘V’ had no effect.
- SEM observation of worn composites confirmed mechanisms of matrix wear, fiber pull out, fiber breakage, and debris formation.

## Figures and Tables

**Table 1:** Factors and levels.

Sl. No.	Factors	Values
1	wWt. % of MoS_2_	0, 4, 8, 12
2	‘P’ (N)	5, 10, 15, 20
3	‘V’ (m/s)	1.0, 1.5, 2.0, 2.5
4	‘D’ (m)	500, 750, 1000, 1250

**Table 2:** L16 experiments with responses.

Exp. No.	% of MoS2	‘P’ (N)	‘V’ (m/s)	‘D’ (m)	SWR X 10-4 (mm^3^/Nm)	COF	SN Ratio (SWR)	Mean (SWR)	SN Ratio (COF)	Mean (COF)
	(A)	(B)	(C)	(D)						
1	0	5	1.0	500	11.3333	0.60	−18.0874	5.9466	4.4370	0.60
2	0	10	1.5	750	4.5925	0.57	−10.2996	2.5863	4.8825	0.57
3	0	15	2.0	1000	2.7777	0.49	−6.0617	1.6888	6.1961	0.49
4	0	20	2.5	1250	2.3777	0.44	−4.8261	1.5138	7.1309	0.44
5	4	5	1.5	1000	4.9119	0.58	−10.8670	2.7260	4.7314	0.58
6	4	10	1.0	1250	2.0057	0.39	−3.2045	1.2028	8.1787	0.39
7	4	15	2.5	500	3.8886	0.55	−8.8857	2.2410	5.1927	0.55
8	4	20	2.0	750	2.3536	0.43	−4.6163	1.4268	7.3306	0.43
9	8	5	2.0	1250	3.2625	0.52	−7.3869	1.9112	5.6799	0.52
10	8	10	2.5	1000	1.9442	0.70	−3.0428	1.2221	3.0980	0.70
11	8	15	1.0	750	1.6860	0.35	−1.7649	1.0430	9.1186	0.35
12	8	20	1.5	500	2.6081	0.46	−5.4436	1.5290	6.7448	0.46
13	12	5	2.5	750	2.0263	0.40	−3.2181	1.1631	7.9588	0.40
14	12	10	2.0	500	2.8875	0.50	−6.2828	1.6437	6.0206	0.50
15	12	15	1.5	1250	0.8308	0.32	3.9721	0.5820	9.8970	0.32
16	12	20	1.0	1000	0.8168	0.30	4.2178	0.5584	10.4576	0.30

**Table 3:** Response for signal-to-noise ratios (smaller-is-better criterion).

Level	Wt. % of MoS_2_	‘P’ (N)	‘V’ (m/s)	‘D’ (m)
1	−9.8187	−9.8899	−4.7098	−9.6749
2	−6.8934	−5.7074	−5.6595	−4.9747
3	−4.4096	−3.1850	−6.0869	−3.9384
4	−0.3278	−2.6671	−4.9932	−2.8613
Delta	9.4909	7.2228	1.3771	6.8135
Rank	1	2	4	3

**Table 4:** Response table for means.

Level	Wt. % of MoS_2_	‘P’ (N)	‘V’ (m/s)	‘D’ (m)
1	2.9339	2.9368	2.1877	2.8401
2	1.8992	1.6638	1.8559	1.5548
3	1.4264	1.3887	1.6677	1.5489
4	0.9869	1.2571	1.5350	1.3025
Delta	1.9471	1.6797	0.6527	1.5376
Rank	1	2	4	3

**Table 5:** Response table for signal-to-noise ratios for COF.

Level	wt. % of MoS_2_	‘P’ (N)	‘V’ (m/s)	‘D’ (m)
1	5.662	5.702	8.048	5.599
2	6.358	5.545	6.564	7.323
3	6.160	7.601	6.307	6.121
4	8.583	7.916	5.845	7.722
Delta	2.922	2.371	2.203	2.123
Rank	1	2	3	4

**Table 6:** Response table for means for COF.

Level	wt. % of MoS_2_	‘P’ (N)	‘V’ (m/s)	‘D’ (m)
1	0.5250	0.5250	0.4100	0.5275
2	0.4875	0.5400	0.4825	0.4375
3	0.5075	0.4275	0.4850	0.5175
4	0.3800	0.4075	0.5225	0.4175
Delta	0.1450	0.1325	0.1125	0.1100
Rank	1	2	3	4

**Table 7:** Analysis of variance for SWR.

Source	DF	Adj SS	Adj MS	F-Value	*p*-Value
Wt. % of MoS_2_	3	29.579	9.860	4.62	0.120
‘P’ (N)	3	28.151	9.384	4.40	0.127
‘V’ (m/s)	3	4.487	1.496	0.70	0.611
‘D’ (m)	3	22.821	7.607	3.57	0.162
Error	3	6.396	2.132		
Total	15	91.433			

**Table 8:** Analysis of variance for COF.

Source	DF	Adj SS	Adj MS	F-Value	*p*-Value
Wt. % of MoS_2_	3	0.05095	0.016983	4.03	0.141
‘P’ (N)	3	0.05415	0.018050	4.28	0.132
‘V’ (m/s)	3	0.02655	0.008850	2.10	0.279
‘D’ (m)	3	0.03710	0.012367	2.93	0.200
Error	3	0.01265	0.004217		
Total	15	0.18140			

## Data Availability

The original contributions presented in this study are included in the article; further inquiries can be directed to the corresponding author.
